# The multifaceted roles of mucins family in lung cancer: from prognostic biomarkers to promising targets

**DOI:** 10.3389/fimmu.2025.1608140

**Published:** 2025-06-27

**Authors:** Qinghong Yuan, Sirui Cai, Yuanhang Chang, Junqi Zhang, Meng Wang, Kun Yang, Dongbo Jiang

**Affiliations:** ^1^ Department of Immunology, Basic Medicine School, Air-Force Medical University (The Fourth Military Medical University), Xi’an, China; ^2^ The Key Laboratory of Bio-Hazard Damage and Prevention Medicine, Basic Medicine School, Air-Force Medical University (The Fourth Military Medical University), Xi’an, China; ^3^ Department of Rehabilitation Medicine, Tangdu Hospital, Air-Force Medical University (The Fourth Military Medical University), Xi’an, China

**Keywords:** mucins, lung cancer, biomarkers, targets, prognosis, therapeutics

## Abstract

Lung cancer remains one of the leading causes of cancer-related deaths worldwide, highlighting the urgent need for enhanced diagnostic and therapeutic strategies. Mucins, a family of glycoproteins crucial for maintaining epithelial integrity and regulating immune responses, have emerged as promising biomarkers and therapeutic targets in the context of lung cancer. The expression patterns and functional roles of mucin family members significantly influence lung cancer progression, thereby shaping diagnostic and therapeutic approaches for this disease. A more detailed classification of mucin family members could facilitate diagnosis and patient assessments, as well as help identify potential therapeutic targets. This review thoroughly examines the latest advancements in understanding the role of mucins in lung cancer progression, prognosis, and treatment, while also highlighting knowledge gaps and opportunities for future research, thus providing new perspectives for the management of this disease.

## 1 Introduction

Lung cancers, the primary causes of cancer-related deaths globally, are a group of malignancies that develop from bronchial epithelial cells (1). According to data from the International Agency for Research on Cancer (IARC) of World Health Organization, the incidence of lung cancer is increasing annually, particularly in developing nations (2). Despite significant progress in diagnosing and treating lung cancers, the five-year survival rate after diagnosis remains less than desirable (3–5). The main difficulty lies in detecting lung cancer early, as it frequently advances to a late stage before patients are diagnosed (6).

### 1.1 Classification and epidemiology

They can be histologically categorized into non-small cell lung cancer (NSCLC) and small cell lung cancer (SCLC). Notably, NSCLC accounts for more than 85% of cases and includes adenocarcinoma, squamous cell carcinoma, large cell carcinoma, among others. SCLC, although uncommon, is the most aggressive pulmonary neoplasm, characterized by a rapid doubling time and the development of widespread metastases (7). The clinical presentation of lung cancer is varied and may include common symptoms such as coughing, chest discomfort, difficulty breathing, weight loss, and abnormal blood collection (8).

### 1.2 Current treatment approaches and research hotspots

The incidence of lung cancer has increased due to the acceleration of industrialization and changes in lifestyle. The etiology of lung cancer is complex, with smoking being identified as the most significant risk factor (9). Long-term smoking is directly associated with an increased likelihood of developing lung cancer (10). Additionally, factors such as second-hand smoke exposure, air pollution, occupational exposure (for instance, asbestos and uranium mining), genetic susceptibility, and certain hereditary diseases also contribute to the risk of developing lung cancer (11).

Current treatment regimens for lung cancer include surgery, chemotherapy, radiotherapy, and targeted therapy. Surgical resection offers the best chance of cure for early-stage NSCLC, while chemotherapy and radiotherapy are used for locally advanced and metastatic disease.

Some of the latest anti-cancer treatments include immune checkpoint inhibitors (ICIs), which have been proven to have a high possibility to act as specific targets for successful treatment, especially for NSCLC (12). Despite our ability to use predictive biomarkers such as PD-L1 (Programmed cell death ligand 1) expression and a high TMB (tumor mutational burden) as indicators for ICIs treatment, the widespread clinical use of ICIs is limited by various constraints (13, 14). Hence, researchers are continuously trying to find other early, effective, and minimally invasive approaches or advanced screening tools that might benefit immunotherapy.

Biomarkers for lung cancer have been studied in recent years, and the remarkable findings have enhanced the accuracy of clinical diagnoses and treatments. These biomarkers have not only enhanced disease diagnoses and typing but also guided differential treatment regimens and the evaluation of responses to treatments and prognoses, particularly in immunotherapy. Biomarkers are critical to understanding treatment response, monitoring the effectiveness follow-up, identifying resistance mechanisms, and identifying new treatment targets (15).

Recent advances have underscored the mucin family possessing profound functional and structural significance. By elucidating their shared characteristics (summarized in **Table 1**), this review establishes a robust framework for dissecting their distinct mechanistic pathways and roles in diverse biological contexts. This structured approach not only enhances our understanding of mucins but also emphasizes their translational potential as targets for therapeutic innovation.

**Table 1:** Description of the common characteristics of the mucin family.

Common characteristics	Description
Structural Features	Members of the mucin family typically have high molecular weights and are rich in O - glycosylation.
Tandem Repeat Sequences	Most mucins contain tandem repeat sequences, which are rich in proline, threonine, and serine (PTS domains), an important feature of mucins.
Secretory and Membrane - Bound Types	The mucin family can be divided into secretory types (such as MUC2, MUC5AC, MUC5B, MUC6) and membrane - bound types (such as MUC1, MUC3A, MUC4, MUC16).
Cellular Localization	Secretory mucins are mainly located extracellularly, forming a mucus layer; membrane - bound mucins are anchored to the cell membrane and participate in cell signaling.
Functions	1. Protect and lubricate the epithelial surface. 2. Maintain epithelial integrity and participate in cell adhesion. 3. In tumorigenesis, the abnormal expression of mucins is associated with the occurrence, development, and prognosis of various cancers.
Roles in Tumors	1. Promote the proliferation and invasion of tumor cells. 2. Transmit carcinogenic signals through interactions with growth factor receptors. 3. Have anti - apoptotic effects, protecting cancer cells from the effects of chemotherapy.
Expression Patterns	The expression patterns of mucins differ between normal and tumor tissues. Changes in their expression levels and subtypes are closely related to the biological behavior of tumors.
Evolutionary Diversity	The mucin family shows a high degree of diversity during evolution, with different members having different structural domains and functions.

### 1.3 Focus on mucins

Mucins are high-molecular-weight glycoproteins that play essential roles in maintaining physiological homeostasis across various tissues, particularly in the respiratory tract, where they serve as protective barriers against environmental insults (16). However, in lung cancer, their dysregulation and overexpression are critical factors influencing disease initiation, progression, and metastasis (17).

In physiological settings, mucins serve as vital components of the mucosal defense system, safeguarding epithelial surfaces against pathogens and toxins. These transmembrane glycoproteins are predominantly expressed in epithelial cells and are crucial for maintaining cell integrity and surface smoothness (18). Their primary function throughout the body is to form protective barriers, playing a pivotal role in preserving mucosal integrity, mitigating inflammatory cell infiltration, maintaining alveolar surface hydration, and modulating the phagocytic efficiency of alveolar phagocytes (19–21).

For instance, MUC5B, a major mucin in the airways, plays a crucial role in sustaining mucociliary clearance, which is an essential defense mechanism against inhaled pathogens and pollutants. Its expression can be upregulated during lung injury to modulate inflammatory responses, thereby reducing the secretion of inflammatory factors, promoting goblet cell differentiation, and limiting excessive inflammation. This demonstrates its protective role in lung diseases such as chronic obstructive pulmonary disease (COPD) and idiopathic pulmonary fibrosis (IPF) (22–25).

Chronic inflammatory states, such as those induced by cigarette smoke exposure, can lead to sustained overexpression of mucins. This overexpression may ultimately predispose cells to malignancy, as elevated levels of MUC1 in response to chronic inflammation have been correlated with an increased risk of carcinoma. This observation demonstrates how a protective mechanism can shift toward promoting carcinogenesis under pathological conditions (26, 27).

Abnormal mucin expression can lead to alterations in cell adhesion, invasion, and metastasis, which are critical processes in cancer development and dissemination. Some mucins also modulate signaling pathways, thereby enhancing tumor survival and resistance to treatment (16, 17). For instance, cell surface mucins such as MUC1 initially serve as a protective barrier against pathogen invasion by acting as both a physical wall and a decoy for microbial binding. However, prolonged or chronic infections may induce persistent inflammation via MUC1-mediated signaling. MUC1 interacts with TLR and NLRP3 inflammasome pathways: while it can initially suppress excessive inflammation by competitively inhibiting MyD88/TRIF recruitment to TLRs, sustained interactions with microbes may exacerbate pro-inflammatory responses, ultimately fostering a tumor-promoting microenvironment (28, 29).

Mucins, such as MUC3A, have been identified not only as markers of poor prognosis but also as active regulators of key signaling pathways that promote cancer development. In NSCLC, elevated levels of MUC3A correlate with increased tumor growth, reduced sensitivity to therapies such as radiation, and the upregulation of immune checkpoint proteins like PD-L1, which facilitate immune evasion by tumors (30, 31).

In summary, mucins exhibit a dualistic role in lung tissue: they provide critical protective functions against pathogens and maintain lung homeostasis; however, their dysregulation drives cancer-associated pathological processes. Deciphering this balance is pivotal for developing therapeutic strategies that selectively mitigate oncogenic mucin activity while preserving their protective roles in lung health. Mucins hold promise as biomarkers to overcome current limitations in lung cancer diagnostics and therapeutics. Their utility in early detection, accurate tumor typing, and patient stratification—by virtue of cancer-type-specific expression patterns—may guide the development of minimally invasive or individualized therapies. In this context, mucin biomarkers represent a promising frontier for advancing precision oncology in lung cancer and improving clinical outcomes.

A concise review would be necessary on explicating mucins’ dual roles in lung cancer, detailing their expression, functions, and clinical applications. By synthesizing these findings, we provide novel insights to reframe perspectives on the complex interplay between mucins and lung cancer, thereby paving the way for next-step exploitation on this challenging disease.

## 2 The mucin family as biomarkers

Molecular markers, including genetic mutations, proteins, and other molecular indicators, have revolutionized the practice of oncology through early diagnoses and cancer treatments (32–34). These biomarkers not only help in diagnosing cancer in its earliest stages but are also highly useful in determining appropriate clinical responses to the cancer via different therapies. Moreover, they are helpful for evaluating the extent of the cancer and the progress of the patients during their subsequent treatments and checkups.

### 2.1 Classification of mucins

Understanding mucins’ functions and structures is thus critical to diagnosis and treatment (35). Mucins can be categorized into two groups: membrane-bound mucins and secretory (or gel-forming) mucins (**Table 2**), which are both types of mucin molecules (18). Membrane-bound mucins are predominantly located on the surface of epithelial cells. They typically exhibit a single-chain structure that traverses the cell membrane and possesses an extracellular domain that is extensively O-glycosylated (36). Secreted mucins are primarily synthesized by goblet cells and certain glandular epithelial cells. These mucins are distinguished by their extensive glycosylation and their capacity to form large, gel-like structures upon secretion, which are essential for the viscosity and protective functions of mucus (37, 38). Both types possess the ability to form protective shields that are effective against toxic substances and microorganisms potentially damaging the mucosa.

**Table 2:** Chromosomal localization and tissue expression of the mucin gene.

Gene symbol	Chromosome band	Entrez gene ID	Mucin form	Expression section
MUC1	1q22	4582	Membrane-bound mucin	Lung tissue
MUC2	11p15.5	4583	Secreted	Goblet cell
MUC3	7q22	4584/57876	Membrane-bound mucin	Lung tissue
MUC4	3q29	4585	Membrane-bound mucin	Lung tissue
MUC5	11p15.5	4586/727897	Secreted	Lung tissue
MUC6	11p15.5	4588	Secreted	Gastrointestinal tissue
MUC7	4q13.3	4589	Secreted	Salivary gland tissue
MUC8	12q24.33	100129528	Secreted	Lung tissue
MUC9	1p13.2	5016	Secreted	Lung tissue
MUC12	7q22.1	10071	Membrane-bound mucin	Gastrointestinal tissue
MUC13	3q21.2	56667	Membrane-bound mucin	Gastrointestinal tissue
MUC14	4q24	51705	Membrane-bound mucin	Lung tissue
MUC15	11p14.2	143662	Membrane-bound mucin	Lung tissue
MUC16	19p13.2	94025	Membrane-bound mucin	Lung tissue
MUC17	7q22.1	140453	Membrane-bound mucin	Intestinal tissue
MUC18	11q23.3	4162	Membrane-bound mucin	Lung tissue
MUC19	12q12	283463	Secreted	Salivary gland tissue
MUC20	3q29	200958	Membrane-bound mucin	Kidney tissue
MUC21	6p21.33	394263	Membrane-bound mucin	Lung tissue
MUC22	6p21.33	100507679	Membrane-bound mucin	Lung tissue
MUC24	6q21	8763	Membrane-bound mucin	Lung tissue

Despite the similarities in their roles, the synthesis of mucins exhibits notable distinctions in localization, composition, and regulation within the body (39). For instance, MUC1 is located on chromosome 1q21, whereas MUC4 is found on chromosome 3q29 (40, 41). Membrane-bound mucins play a crucial role in regulating hydration at mucosal surfaces, contribute to immune responses, and facilitate cellular interactions with the external environment. Their aberrant expression is frequently associated with various diseases, particularly cancers, underscoring their importance in both health and disease contexts (42–44). The functional significance of secreted mucins extends beyond mere physical protection; they are integral to the host’s defense mechanisms by trapping pathogens, preventing their adherence to epithelial cells, and coordinating immune responses through interactions with various immune cells. Furthermore, the extensive glycosylation of secreted mucins enhances their viscoelastic properties, which are essential for the effective trapping and clearance of pathogens and particles from mucosal surfaces (45, 46).

In shorts, both types of mucins play important roles in protecting the body from harmful substances, but dysregulation can contribute to negative diseases outcomes such as cancer or inflammation (47–50). Understanding the classification and roles of mucins is essential for deciphering the complexities of mucosal immunity, disease mechanisms, and potential therapeutic targets, particularly in relation to respiratory system-related diseases. Insights into mucin biology may lead to novel strategies aimed at modulating mucin production or function, which could have significant implications for the treatment of various pathologies.

### 2.2 Mucin expression in lung cancer

Members of the mucin family demonstrate varying levels of expression in lung tissue. Specifically, MUC1, MUC2, MUC3A, MUC3B, MUC4, MUC5AC, MUC5B, MUC6, MUC7, MUC8, MUC11, MUC12, MUC13, MUC15, MUC16, MUC17, MUC19, MUC20, MUC21, and MUC22 have all been identified in lung tissue (51–54). In specific, study reported that tumor cells showed expression levels of MUC1 (77%), MUC2 (2%), MUC5B (63%), MUC5AC (36%) and MUC6 (21%) (55).

#### 2.2.1 Expression patterns of mucins in lung cancer

The expression patterns of mucins in lung cancer tissues exhibit considerable diversity and significantly influence disease progression. Notably, the expression levels of MUC1, MUC2, MUC3A, MUC16, and MUC17 in NSCLC have been shown to correlate with patient prognosis. MUC5AC is predominantly expressed in the trachea and main bronchi, but is absent in bronchioles and smaller alveolar epithelial cells. In contrast, MUC5B is detected in the bronchial epithelium, mucus-producing cells, and glandular ducts, while it is not expressed in bronchiolar or alveolar epithelial cells.

Membrane-bound glycoprotein, is recognized for its role in promoting tumor progression and modulating immune responses. It has been implicated in enhancing tumor invasiveness and protecting cancer cells from immune destruction. The upregulation of MUC1 is a hallmark of lung cancer, particularly in NSCLC, with its expression level closely associated with tumor aggressiveness, dissemination, and recurrence (51). The altered localization of MUC1, from its normal restricted patterns to a ubiquitous presence on glandular epithelia and within the cytoplasm, underscores its pivotal role in facilitating lung cancer invasion, metastasis, and angiogenesis (52).

The MUC2 expression landscape in lung cancer is intricate, varying significantly across tumor types and stages (56, 57), though it is less frequently encountered compared to gastrointestinal malignancies (58). Meanwhile, MUC4 is a ubiquitous player in multiple tumor types, including pancreatic (59), lung (60), and endometrial cancers (61). In lung cancer specifically, MUC4 expression aligns with histological subtypes, manifesting distinct patterns in NSCLC subtypes like adenocarcinoma, squamous cell carcinoma, and adenosquamous cell carcinoma (62, 63).

On the other hand, MUC5B has been observed to exhibit a contrasting role in lung cancer. It is frequently overexpressed in poorly differentiated adenocarcinomas and has been linked to worse tumor differentiation and disease stage. Studies indicate that while MUC5B serves a protective role in healthy epithelial tissues, its aberrant secretion in lung cancer may promote aggressive cancer behavior, particularly in lung adenocarcinomas (LUAD). High levels of MUC5B not only correlate positively with worse clinical features but are also associated with reduced survival rates, as demonstrated by statistical analyses that employed multivariable models to account for various confounding factors (23).

Further analysis indicates that the interplay between MUC4 and MUC5B expression may hold valuable clinical implications. In lung cancer diagnostics, the combination of MUC4 and MUC5B expression patterns, especially when assessed in conjunction with differentiation markers such as Thyroid Transcription Factor 1 (TTF-1), has been proposed to enhance diagnostic accuracy in distinguishing lung adenocarcinoma from squamous cell carcinoma. This integrated assessment may lead to improved stratification of patients based on aggressiveness and help in tailoring personalized therapeutic strategies (23, 64).

Central to the airway mucin composition, MUC5AC and MUC5B are intimately associated with invasive mucinous adenocarcinoma of the lung (55, 65). MUC5AC’s presence in lung cancer is predictive of tumor aggressiveness and adverse prognosis, correlating directly with malignant features such as lymphatic dissemination, tumor size, and radiographic indicators like lobed and burr signs (66–68). Conversely, Several studies hint at a potential link between MUC5B expression and specific pathological subsets or stages of lung cancer, though definitive conclusions await further validation (69).

MUC5B-AS1, a lncRNA, forms a protective double-stranded structure in lung tissue, increasing the stability of MUC5B mRNA and promoting carcinogenesis. The overexpression of this gene leads to an upregulation of MUC5B expression, as well as the promotion of cell migration and invasion. This is significantly correlated with the Tumor-Node-Metastasis (TNM) stage in lung cancer tissues (24, 70). Meantime, extracellular matrix (ECM) components regulate the production of MUC5B and upregulate it through integrin, extracellular signal-regulated kinase (ERK), and NF-κB-dependent pathways (NF-κB: Nuclear factor kappa B) (20). Additionally, the combined detection of MUC5B and TTF-1 can greatly improve the accuracy of detecting lung adenocarcinoma by distinguishing different cancer cell types (23).

MUC6 is highly expressed in LUAD, especially in CD74–NRG1-rearranged LUAD, and has the ability to inhibit cell proliferation, migration, and invasion (71). At the same time, the inverse correlation between MUC6 and αGlcNAc (N-Acetylglucosamine) may disrupt normal cell communication, while reduced levels of MUC6 can also trigger or worsen inflammatory responses (54).

Notably, MUC16 expression stands out in NSCLC, significantly elevated compared to adjacent non-cancerous tissues, and tightly correlated with clinicopathological parameters like tumor staging, pathological grading, and lymph node involvement (64, 72). MUC16 contains 16 SEA domains in its tandem repeat domain, which potentially, perhaps initiate some of the tumor-related signaling pathways. Additionally, the C-terminal domain of MUC16 is formed of 32 amino acids in the cytoplasmic tail, which may affect the function of the molecule in certain signaling pathways through phosphorylation (73–75).

Additionally, MUC17, which plays a role in signal transduction pathways, helps maintain luminal structure and provides cellular protection in epithelial cells. Furthermore, MUC17 imparts anti-adhesive properties to cancer cells that have lost their polarity and are consequently compromised in their normal cell polarity (76). MUC17 plays a role in maintaining the structural integrity of luminal cells and providing protection for epithelial cells (77).

MUC16 and MUC21 exhibit specific expression patterns and functions in lung cancer. Regarding MUC21, the literature on its expression in lung cancer is scarce, but preliminary findings suggest that its upregulation is associated with diminished infiltration and activation of cytotoxic immune cells, possibly contributing to immune evasion mechanisms in lung cancer (78). Some progress has also been made in the development of a detection antibody related to MUC21 (79).

MUC13, on the other hand, displays elevated levels in LUAD tissues and cells, indicating its potential significance in this tumor type (80, 81). MUC18, also known as MCAM/CD146, is a ubiquitous marker in various tumors, including lung cancer, where it has been implicated in tumor aggressiveness, angiogenesis, and poor prognosis (82–84). Nevertheless, a more exhaustive examination of MUC18’s specific expression patterns in lung cancer tissues, along with its relationship to different tumor types and stages, is warranted.

For other mucins in lung tissue, additional research is imperative to elucidate their specific expressions and functions within lung cancer tissues, contributing to a more comprehensive understanding of the mucin family’s role in lung cancer biology.

#### 2.2.2 Glycosylation of mucins and its impact on lung cancer

The glycosylation of mucins serves as a pivotal regulatory mechanism, influencing not only the biological functions of mucins but also their expression patterns and subcellular distributions. This intricate process ensures that mucins perform their diverse roles efficiently within the physiological landscape (85). For membrane-bound mucins, the glycosylation of mucins can be divided into O-glycosylation and N-glycosylation based on the sites to which the carbohydrate moieties are attached. These forms of glycosylation occur on the extracellular domain of mucins and involve various regulatory factors, such as glycosyltransferases, glycosylases, and transcription factors (TFs), which are crucial for initiating, progressing, and terminating the glycosylation process (86, 87).

The glycosylation status of mucins is critical for their protective functions on epithelial surfaces. For instance, MUC21, a transmembrane mucin, has been demonstrated to protect cells from apoptosis. Transfection of MUC21 into cells leads to resistance against apoptotic signals when it is glycosylated with sialyl T-antigen. This finding indicates that the presence of specific glycan structures, such as sialic acid in the glycoprotein, is essential for its protective function (88).

The glycosylation of mucin has a marked impact on the expression of tumor markers as they are accessible on the cell surface, potentially affecting their signaling and binding to target receptors (87). In lung cancer, aberrant glycosylation results in the expression of tumor-associated carbohydrate structures, notably the Tn antigen. This antigen serves as a hallmark of epithelial tumors and is correlated with increased cancer aggressiveness and poor prognosis. Specifically, the Tn antigen is recognized by the macrophage galactose/GalNAc lectin (MGL), which can modulate immune responses and contribute to the establishment of an immunosuppressive tumor microenvironment (89).

Glycosylation patterns of mucins are not only biomarkers but also potential therapeutic targets. For example, inhibiting O-glycosylation of MUC1 or MUC5AC sensitizes cancer cells to chemotherapy and radiotherapy (18, 67). In lung cancer, targeting glycosyltransferases like GALNT6, which initiates O-glycosylation of mucins, could disrupt mucin-mediated signaling pathways. GALNT6 knockdown in pancreatic cancer cells reduces MUC4 expression and alters cadherin switching from P-cadherin to E-cadherin, suggesting a similar strategy could be effective in lung cancer (90). Furthermore, the glycan-binding properties of mucins can be exploited for diagnostic purposes. For instance, chemical exchange saturation transfer (CEST) MRI detects under-glycosylated MUC1 (uMUC1) in malignant tissues, providing a non-invasive method to assess tumor malignancy (91).

The broader implications of mucin glycosylation in lung cancer extend to immune evasion and microenvironment modulation. Mucin glycosylation has the potential to modify the function of adhesion molecules involved in cell-cell and cell-matrix interactions, which play critical roles in regulating processes such as cell migration, invasion, and metastasis. Additionally, mucin glycosylation may impact tumor growth and metastasis by affecting the ability of tumor cells to evade detection by the immune system.

The overexpression and aberrant glycosylation of mucins can inhibit T-cell interactions, allowing tumor cells to evade immune surveillance. This phenomenon is particularly evident with MUC1, where altered glycosylation can enhance its immunogenicity, leading to modulation of the immune landscape within the tumor (92). In the context of MUC1 and MUC2, MUC1 regulation has been observed during the malignant transformation of cells, resulting in alterations in the glycosylation and localization of the protein, as well as interactions with certain growth factor receptors (93). Sialylated glycans, such as sialyl-Lewis X (sLeX), are overexpressed in lung cancer and interact with selectins on endothelial cells, facilitating metastasis. The binding of sLeX to selectins aids in the adhesion of tumor cells to endothelial cells, thereby promoting the extravasation of tumor cells from the bloodstream and their subsequent metastasis to distant organs (94–96).

Enzymatic remodeling of cell surface glycans, such as the overexpression of β-1,3-glucuronosyltransferase 1 (B3GAT1), has been shown to reduce the expression of sialic acid on the cell surface and inhibit the infection of viruses that rely on sialic acid for cellular entry. Although this mechanism has primarily been explored in virology research, it suggests the potential to modulate cellular functions by altering cell surface glycosylation. In lung cancer research, similar strategies may be explored to block mucin-mediated tumor cell dissemination, thereby providing new insights for the treatment of lung cancer (97). Additionally, glycomic profiling of lung cancer tissues reveals distinct glycosylation signatures, such as increased LacdiNAc and polylactosamine structures, which could serve as prognostic markers or therapeutic targets (98, 99).

Moreover, MUC2 is associated with glycosylation in lung cancer, and its amount is associated with airway inflammation. For patients with stable but severe COPD, MUC2 has been found to play a significant role in airway defense (100).

Overall, the glycosylation status of mucins in lung cancer is not just a marker of disease progression but also plays functional roles in modulating tumor behavior, immune evasion, and resistance to therapies. In the following section, we will delve deeper into the glycosylation of mucins and its impact on lung cancer progression.

#### 2.2.3 Transcriptional regulation of mucins

The regulatory impact of mucin-associated TFs on their expression in lung cancer tissue represents a critical yet underexplored axis in oncogenesis. Emerging evidence highlights their pivotal roles in tumor progression, immune evasion, and therapeutic resistance, with mucins such as MUC1, MUC5AC, and MUC3A—heavily glycosylated proteins governing cellular adhesion, signaling, and barrier function—showing dysregulation in LUAD and lung squamous cell carcinoma (LUSC) that correlates with aggressive phenotypes (101) (102).

For MUC1, its promoter region contains two Sp1, one Sp3, and one E-box (E-MUC1) binding site (103). SP1 directly binds to the MUC1 promoter (-99/-90 locus) to regulate its expression (104), while under hypoxic conditions, HIF-1α interacts with the MUC1 promoter to enhance transcription (105). STAT3 acts as an upstream regulator of MUC1, modulating its transcription via the JAK/STAT signaling pathway (106). Additionally, DPP9 regulates MUC1 expression at both the mRNA and protein levels (107). For MUC4, PRDM16-ΔPRD influences its transcription by modifying histone acetylation at the MUC4 promoter (108).

The MUC5B promoter harbors enhancer elements regulated by STAT3 and SPDEF, which bind to specific sites to activate transcription. Notably, the MUC5B enhancer region in lung cancer cells exhibits chromatin accessibility, with RNA polymerase II loading near the key SNP rs35705950—indicating a direct role for this variant in enhancer function (109). This regulatory network is further complicated by nuclear receptors such as the vitamin D receptor (VDR) and retinoic X receptor (RXR), which may modulate mucin gene expression via heterodimeric interactions (30, 31, 69, 109). The MUC5B promoter variant rs35705950 (a G/T transversion), located within a −3 kb enhancer region, undergoes chromatin accessibility and epigenetic reprogramming in idiopathic pulmonary fibrosis (IPF)—a disease linked to lung cancer risk. This enhancer recruits RNA polymerase II alongside TFs such as FOXA2 and XBP1, which bind near the variant in a genotype-dependent manner to regulate MUC5B expression (109–111). XBP1, a mediator of endoplasmic reticulum stress, directly binds to the −3 kb region, while FOXA2 interacts with a conserved site 32 bp from the variant, underscoring the combinatorial role of TFs in mucin dysregulation (112).

MUC5AC is negatively regulated by the circular RNA circRABL2B. High-throughput sequencing of 141 lung cancer patient samples revealed a significant inverse correlation between circRABL2B and MUC5AC expression, with patients exhibiting low circRABL2B and high MUC5AC levels showing the worst survival outcomes (HR = 2.00; 95% CI = 1.12–3.57). Mechanistically, circRABL2B interacts with the RNA-binding protein YBX1 to suppress MUC5AC transcription, thereby inhibiting the integrin β4/pSrc/p53 signaling axis—a cascade associated with enhanced cellular stemness and chemoresistance. The transcriptional downregulation of circRABL2B, partially mediated by EIF4A3, further elucidates its dysregulation in carcinogenesis (113).

In NSCLC, the transmembrane mucin MUC3A—overexpressed in tumor tissues—is regulated by the NF-κB pathway through its interaction with RELA (p65). MUC3A stabilizes epidermal growth factor receptor (EGFR) and promotes PD-L1 expression via the PI3K/Akt and MAPK signaling pathways, linking mucin-mediated immune evasion to TF activity. Knockdown of MUC3A disrupts RELA phosphorylation and nuclear translocation, attenuating NF-κB activation and its downstream targets (e.g., BRCA-1/RAD51), which are critical for DNA damage repair and radiation resistance. Moreover, the EGF-like domain of MUC3A enhances EGFR signaling, forming a feedforward loop that amplifies mucin-TF interactions during tumor progression (30, 31). These findings highlight TFs as both regulators and effectors of mucin signaling pathways, with profound implications for therapeutic targeting.

The mechanism of action of above TFs offers a foundational framework for elucidating the regulation of mucins in lung cancer. However, more complex regulatory networks and additional potential TFs may be involved, which will be further investigated in the subsequent section. DNA methylation and histone modification are some of the key epigenetic mechanisms that are involved in regulating the expression of mucin genes in cancer epithelial cells. These regulatory processes are critical for inhibiting tumor suppressor genes and promoting oncogenes for the uncontrolled cell growth and development of cancer (86, 114–117).

These findings emphasize the need to understand the changes in post-translational modifications and expression profiles of mucins concerning lung cancer. Studying these mechanisms can provide important information about how complicated the part played by mucins is in lung cancer development, which not only contributes to the availability of new diagnostic and therapeutic approaches but also elucidates the relationship between mucins and lung cancer. Moreover, employing mucins for the biomolecular diagnosis of lung cancer and differentiating between cancer types in patients allows for a more effective treatment plan. Individualizing treatment plans can be a game changer in the diagnosis and treatment of this disease, thus improving the chance of recovery for patients. In light of these studies, more precise and efficient treatments of lung cancer could be provided based on the knowledge of the mechanisms involving mucins.

### 2.3 Clinical applications of mucin biomarkers

Recent studies have demonstrated the promising potential of mucins as cancer biomarkers. Mucin expression levels can be detected in tumor tissues, serum, and other biological fluids using techniques such as immunohistochemistry, ELISA, and mass spectrometry. The use of mucin as biomarker can aid in early detection, tumor typing, and patient stratification, ultimately guiding the selection of appropriate treatment strategies. Recent studies on mucin as a cancer biomarker have been quite encouraging, with investigations showing the ability of mucin to differentiate between various types of cancer based on the degree of expression of these molecules (118). This advancement has led to greater interest in using mucin as a biomarker for diagnosing diseases at the early stages and developing treatments that are tailored to the individual patient. In conclusion, biomarker studies of mucin are fundamental to the improvement of cancer knowledge and control. With reference to the ongoing research (**Table 3**), expectations are high for witnessing significant progress in the early detection of cancer and improved therapeutic interventions using these molecular biomarkers.

**Table 3:** Research hotspots in the treatment of cancer targeting members of the mucin family.

Mucin target	Treatment strategy	Specific drug/technology	Mechanism of action	Clinical trial phase	Indications
MUC1	ADC	BM7 - PE	The anti - MUC1 antibody BM7 is conjugated with Pseudomonas exotoxin A (PE), and the toxin is brought into cancer cells through endocytosis	Phase I/II	Metastatic colorectal cancer
MUC1	ADC	M - 1231	A bispecific antibody - drug conjugate targeting epidermal growth factor receptor (EGFR) and MUC1	Phase I	Various metastatic solid tumors
MUC1	Antibody Therapy	Pab - 001 - MMAE	Targets the extracellular part of MUC1 - C, overcoming the problem of antibody failure caused by the shedding of MUC1 - N subunits	–	Triple - negative breast cancer, etc.
MUC1	ADC	DS - 3939	Targets tumor - specific sialyl - Tn glycoprotein locus (TA - MUC1)	Phase I/II	Various advanced solid tumors (including non - small cell lung cancer, breast cancer, urothelial cancer, ovarian cancer, cholangiocarcinoma, and pancreatic cancer)
MUC1	Bispecific Antibody	PM - CD3 - GEX	Recruits anti - tumor CD3+ T cells to MUC1 - expressing cancer cells	–	–
MUC1	Immunocytokine	PM - IL15 - GEX	Combines interleukin - 15 with PankoMab - GEX to stimulate anti - tumor NK or T cells	–	–
MUC16	ADC	DMUC5754A (sofituzumab vedotin)	Human - derived anti - MUC16 antibody is conjugated with the anti - mitotic agent monomethyl auristatin E (MMAE)	Phase I	Platinum - resistant ovarian cancer, unresectable pancreatic cancer
MUC16	Bispecific T - cell Engager (BiTE)	REGN4018 (MUC16/CD3 BiTE)	Targets MUC16 - positive cancer cells and T cells, exerting an anti - tumor effect	Phase II	Recurrent ovarian cancer
MUC16	BiTE	REGN5668 (MUC16/CD28 BiTE)	Targets MUC16 - positive cancer cells and CD28+ T cells	Phase II	Recurrent ovarian cancer
MUC16	Chimeric Antigen Receptor T - cell (CAR - T) Therapy	JCAR - 020	Targets MUC16 and carries interleukin - 12 receptor agonist	Phase I	Recurrent ovarian cancer
MUC16	Bispecific Antibody	LBL - 033	Targets MUC16 and CD3, recruiting T cells to attack cancer cells	Phase I/II	Various tumors
MUC16	Bispecific Antibody	Ubamatamab	Targets MUC16 and CD3, recruiting T cells to attack cancer cells	Phase II	Ovarian cancer, peritoneal cancer, fallopian tube cancer, and endometrial cancer
MUC13	Antibody Therapy	–	Targets MUC13 for the diagnosis and treatment of pancreatic cancer	–	Pancreatic cancer
MUC17	Bispecific T - cell Engager (BiTE)	AMG 211	Targets CD3 and MUC17 for the treatment of gastric cancer and esophageal cancer	Phase I	Gastric cancer, esophageal cancer

Furthermore, advancements in technology have led to the application of even more precise and intricate techniques in the detection of mucin biomarkers; for instance, liquid biopsy or imaging techniques. These innovations have also contributed to the development of mucin as a biomarker and provide a tremendous opportunity to improve the quality of life for oncology patients (119).

## 3 Significance of research on mucins in lung cancer

The role of the mucins in lung cancer has been a long topic of great importance for clinical practice. By understanding the complex associations between mucins and lung cancer, the fields can come up with better ways of determining the causative factors of this deadly disease, which in turn help to identify novel biomarkers for early diagnosis, new targets for treatment, and promising treatment regimens. Furthermore, a deeper understanding of the role of mucins could help in constructing more accurate prognostic markers, which will be important to individualized treatment plans and patient survival (86, 120).

### 3.1 Role of mucins in the occurrence and development of lung cancer

**Figure 1:**
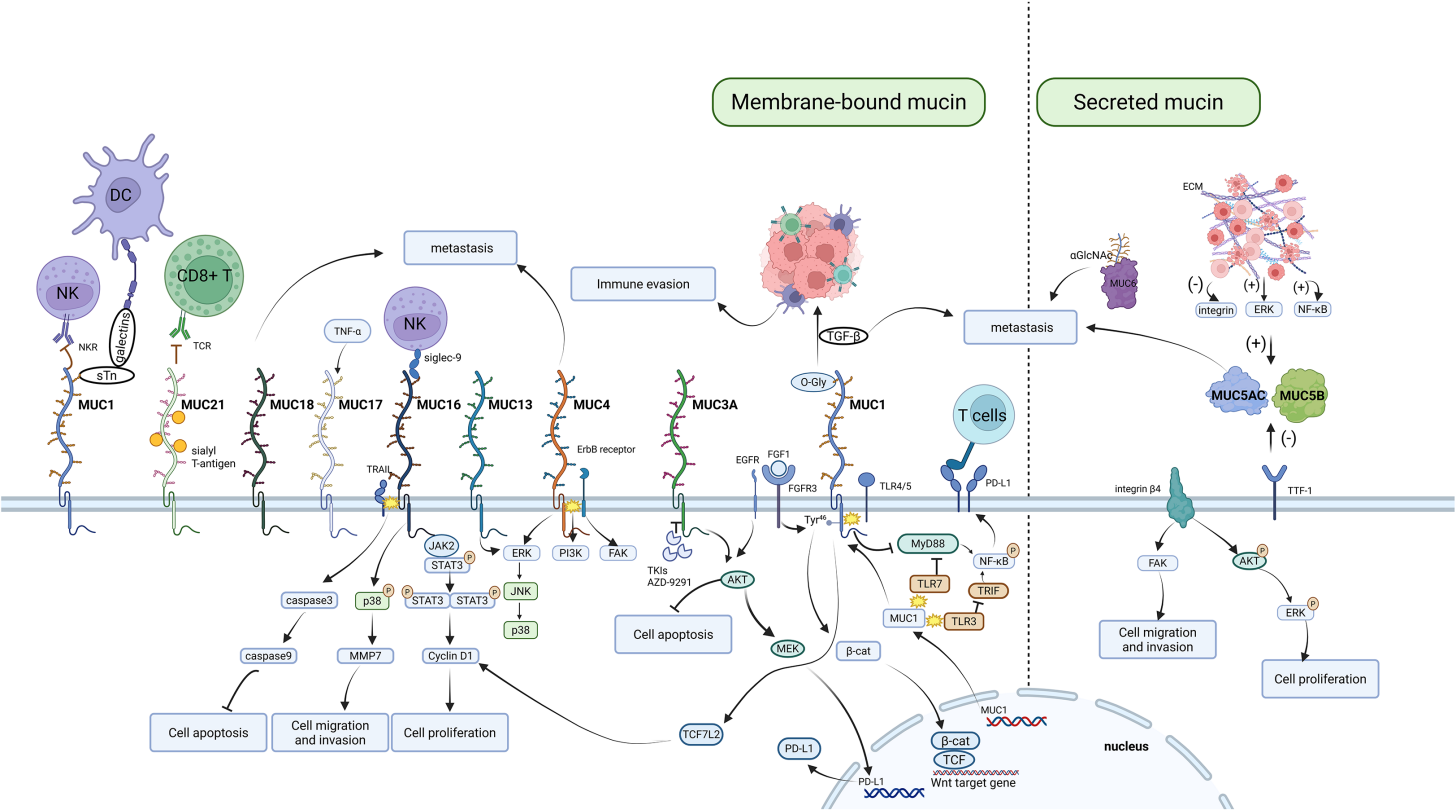
Mechanistic map of aberrantly expressed mucin family members driving cancer progression. Focuses on the mucin functional network, illustrating their biological roles in supporting tumor progression through mechanisms such as disrupting immune responses, activating signaling pathways, regulating apoptosis and proliferation, promoting cell migration and invasion, and interacting with the extracellular matrix.

At present, studies on the mucin family are still in the early stages, and the role of mucins in the initiation and progression of lung cancer is extremely complex. We thus categorize the impact of mucins on lung cancer into several key processes (**Figure 1**):

1. Signal transduction: Mucins can interact with various signaling proteins, such as kinases and TFs, impacting downstream signaling pathways associated with tumor growth (121).
2. Cell proliferation: Some mucins, like MUC1, can facilitate the release of growth factors and prostaglandins, thereby promoting cell proliferation (122).
3. Apoptosis: Certain mucins, such as MUC4 and MUC21, have been shown to inhibit apoptosis and to promote tumor cell survival (123, 124).
4. Migration and invasion: Some mucins can regulate cell adhesion and motility via epithelial - mesenchymal transition (EMT) (123, 125).
5. Immune evasion: The altered glycosylation of mucins can help tumor cells avoid immune detection (126).

The illustration of several key members of the mucin family is expanded in the following sections.

The intricate structure of MUC1 underscores its significance in medical research and clinical assessment. In normal tissues, MUC1 assumes a branched architecture, adorned with polysaccharides, enhancing its surface area for interactions with the extracellular environment. It primarily functions as a protective shield, lubricating agent, and structural stabilizer, while also engaging in cellular signaling and recognition processes (127–130). Its structure encompasses: Amino Acid Sequence: Arranged in 5 repetitive units, each containing approximately 20 amino acid residues, contributing to its unique composition; Glycan Chain Composition: Rich in mannose and galactose, intricately linked via both N-linked and O-linked glycosidic bonds, adding to its complexity; Functional Domains: Comprising a central repetitive region, along with distinct C-terminal and N-terminal regions, each with specialized biological activities; Expression Regulation: Subjected to intricate control by hormones, growth factors, and tumor-associated signaling pathways, highlighting its responsiveness to environmental cues. MUC1-C (transmembrane-cytoplasmic segment) consists of an extracellular domain of 58 amino acids, a transmembrane domain of 28 amino acids, and a cytoplasmic tail region of 72 amino acids. Its cytoplasmic tail region contains multiple phosphorylation sites (such as Tyr and Ser residues) and can interact with signaling pathways such as EGFR, PI3K/AKT, and β-catenin. Under normal physiological conditions, MUC1-N and MUC1-C form a heterodimer through non-covalent bonds and are localized to the apical membrane of the cell; in a tumor environment, MUC1-N is prone to shedding, while MUC1-C remains continuously activated and undergoes internalization (131–133).

In diseased tissues, particularly cancer, can lead to alterations in MUC1 expression and structure, manifesting as overexpression and formation of a dense membrane layer. These changes may hinder drug penetration and disrupt normal physiological functions. In disease states, MUC1’s role shifts toward facilitating cancer cell growth, metastasis, and invasion, while simultaneously diminishing the responsiveness of cancer cells to chemotherapy and radiotherapy (134, 135). And its special structure is precisely the main mechanism by which MUC1 promotes anaplastic apoptosis resistance. MUC1’s extensive O-glycosylation facilitates its interactions with various cell surface molecules that initiate anoikis. By influencing the cell surface environment, MUC1 prevents the engagement of these anoikis-initiating signals that would normally trigger cell death when anchorage to the ECM is lost. Specifically, studies have shown that the suppression of Core 1Gal-transferase (C1GT), which leads to significant reductions in MUC1 O-glycosylation, results in increased susceptibility to anoikis in MUC1-positive cancer cells. This effect is linked to enhanced accessibility to critical ligands on the cell surface, such as E-cadherin and integrin β1, which initiate the anoikis process. Furthermore, MUC1’s capability to create a supportive microenvironment enables cancer cells to evade the initial signals that would otherwise activate apoptotic pathways. Consequently, when cancer cells detach, MUC1 aids in sustaining signals that promote survival pathways, ultimately enhancing their metastatic potential (136–138).

The cytoplasmic domain of MUC1 can physically associate with numerous signal transduction proteins with kinase activity such as PKCα (Protein Kinase C Alpha), PKCδ (Protein Kinase C Delta), GSK3β (Glycogen Synthase Kinase 3 Beta), EGFR, and cSrC (a proto-oncogene). It also binds to nonkinase active signal molecules, including p53 (a tumor suppressor gene), Erα (Estrogen receptor α), and β-catenin, although they are not kinases. These interactions engage signaling processes relevant to tumor formation (121).

MUC1 interacts closely with EGFR, which promotes cancer progression and influences therapeutic responses, significantly impacting the pathology NSCLC. Animal model studies have demonstrated that MUC1 can enhance carcinogen-induced EGFR activation in lung bronchial epithelial cells, indicating that MUC1 may lead to lung carcinogenesis by stabilizing activated EGFR. This stabilization enhances cellular signaling pathways that contribute to tumor growth and survival. Consequently, prolonged EGFR activity, which is frequently upregulated in various lung cancers, drives proliferation and metastasis (139).

In the context of chemotherapy, particularly with paclitaxel-based drugs, the expression of MUC1 is closely associated with the drug resistance mechanism in lung adenocarcinoma. Research has demonstrated that high-level expression of MUC1, through its involvement in the NF-κB and MAPK signaling pathways, could activate the EGFR signaling cascade, which linked to cancer stem cell characteristics. This activation may contribute to maintaining a chemo-resistant cell population. Furthermore, the interaction between MUC1 and EGFR may partially promote drug resistance by activating the IL-6 signaling pathway, which is implicated in cancer stem cell enrichment and subsequent chemotherapy failure (140).

The regulatory dynamics between MUC1 and EGFR involve multiple signal transduction pathways. Clinical and cellular studies indicate that MUC1 overexpression enhances the stability of EGFR and promotes ligand-independent activation, resulting in the upregulation of downstream targets in the PI3K/Akt pathway, including phosphorylated AKT and mTOR (56, 141). This upstream regulation of EGFR signaling by MUC1 establishes a pro-proliferative microenvironment, which is distinct from the downstream effects of MUC1-C on PI3K/Akt described in other research (56, 141).

MUC1 has been shown to play a pivotal role in inducing prostaglandin and growth factor synthesis, which are crucial for promoting cancer cell proliferation and survival. However, the precise molecular mechanisms underlying these interactions remain unclear, necessitating further investigation (93). The molecular mechanism of oncogenic signaling is the phosphorylation of the cytoplasmic tail of MUC1, which then acts as a receptor, binds to TFs, and translocates into the nucleus to activate further signaling pathways (142). On the whole, MUC1 has a significant function in signal transduction throughout the carcinogenic process and is strongly associated with EMT, invasion, metastasis, and chemoresistance (122).

The C-terminal domain of MUC1, formerly described as MUC1-C, has a disordered structure, while the cytoplasmic domain of the CQCRRK sequence interacts with the cytoplasmic membrane (143). In patients with NSCLC, an excessive expression of MUC1-C is linked to the downregulation of genes related to immunosuppression and a poor prognosis (144). As a cancer-related protein, MUC1-C exerts its influence on downstream signaling pathways by interacting with a variety of signaling molecules. For instance, it activates the transcription factor TCF7L2, thereby promoting the expression of cyclin D1 (145). In addition, MUC1-C can be targeted to mitochondria through its interaction with c-Src and the molecular chaperone HSP90 (146).

MUC1-C serves as a common mediator of osimertinib resistance in NSCLC. Targeting MUC1-C has been shown to reverse osimertinib resistance in various models (147). It also promotes angiogenesis by upregulating vascular endothelial growth factor (VEGF) expression, thereby providing nutrients for tumor growth (148). At the same time, MUC1 is involved in regulating the tumor microenvironment by modulating the expression of CD274/PD-L1. Studies indicate that MUC1-C directly activates PD-L1 expression in lung cancer cells, thereby contributing to immune evasion (21). MUC1-C achieves this by increasing NF-κB p65 occupancy on the CD274/PD-L1 promoter, which enhances CD274 transcription and further suppresses the activity of immune effectors such as TLR9 and IFNG. This interplay suggests a feedback loop in which MUC1 not only inhibits immune responses but also actively promotes pathways that protect the tumor from immune clearance, a common characteristic of malignant cells in lung cancer (149).

Altogether, MUC1-C has been observed to induce expression of inflammatory cytokines, promoting a supportive environment for tumor growth and survival. By influencing the activation of immune cells within the tumor microenvironment, MUC1-C also assists in establishing a pro-tumorigenic milieu that can help tumors evade immune surveillance (150).

Importantly, MUC1-C’s role extends to the modulation of apoptotic signals in lung cancer cells. By interfering with apoptotic pathways, MUC1-C can protect tumor cells from chemotherapy-induced death, contributing to the overall resistance of lung cancer to treatment. This protective mechanism illustrates why targeting MUC1-C could have therapeutic implications, particularly in enhancing the efficacy of existing lung cancer treatments (106, 151). Based on the above, combining therapies that target MUC1-C with traditional chemotherapy or immunotherapy could enhance treatment efficacy against lung cancer. Such strategies have the potential to disrupt the functions of MUC1-C, restore immune responses against tumors, and circumvent resistance mechanisms that complicate lung cancer management (147).

In paclitaxel-resistant NSCLC cells, the expression of MUC1-C is upregulated, correlating with the activation of the PI3K/Akt signaling pathway. Inhibition of MUC1-C can reverse this resistance. In SCLC, MUC1 interacts with PP2A to enhance its activity, thereby inhibiting the activity of PKCζ. This ultimately reduces the phosphorylation of NUMB, promoting the symmetric division and expansion of cancer stem-like cells (CSCs), which contributes to chemoresistance (152). Silencing MUC1-C in H1975 cells carrying the EGFR driver mutation L858R/T790M can inhibit lung cancer cell proliferation by suppressing the protein kinase B (Akt) signaling pathway. The MUC1 inhibitors GO-201, -202, and -203 can directly bind to the MUC1 cytoplasmic domain and slow MUC1-induced cell proliferation. GO-203, in combination with afatinib, can inhibit the growth of NSCLC cells with EGFR (T790M) or EGFR (delE746-A750) mutations. MUC1 can also interact with estrogen receptors α and β in the nucleus to inhibit the proliferation of LUAD cells (143).

Phosphorylation of the MUC1 cytoplasmic tail is a pivotal step in its oncogenic signaling. Once phosphorylated, MUC1 acts as a receptor that binds to TFs and translocates to the nucleus to activate downstream signaling pathways (153).After being phosphorylated by tyrosine kinases such as EGFR, MUC1-C can directly bind to the SH2 domain of PI3K, activate the AKT→mTOR signaling pathway, and also stimulate the MEK/ERK signaling pathway (154). The RAS-associated domain family IA (RASSF1A) is an inhibitor of the MEK/ERK signaling pathway. Inhibiting the expression of RASSF1A would indirectly promote the activation of the MEK/ERK signaling pathway (16). In addition, the MEK/ERK signaling pathway is also involved in the PD-L1 expression process induced by MUC1-C, thereby affecting the immune escape of the tumor (155).

MUC1 interacts with various cells in the TME, including tumor-associated macrophages (TAMs), which are known to promote cancer growth and metastasis through the secretion of growth factors and cytokines. Research indicates that MUC1 expression is upregulated in TAMs, particularly in the M2 phenotype, which is associated with inflammation and tumor progression. The presence of MUC1 in this context can enhance cancer stem cell properties and contribute to the inflammatory milieu that supports tumor growth (156).

Moreover, MUC1 has been linked to the secretion of tumor necrosis factor-alpha (TNFα) from macrophages. This cytokine promotes inflammatory conditions that are conducive to cancer development. Additionally, MUC1 can regulate the differentiation and function of immune cells, influencing the immune response in the TME. For instance, high levels of MUC1 may suppress cytotoxic T cell activity, thereby aiding tumor escape from immune surveillance (156, 157). Therefore, MUC1-C therapy may enhance the activation and killing ability of CD8(+) T cells by activating the immune microenvironment (17). Besides, MUC1 influences the uptake ability of dendritic cells (DCs) (158).

Notably, a study has revealed that higher expression of MUC1 can potentiate the tumor-promoting functions of TGF-β. In the tumor microenvironment, MUC1 may cooperate with TGF-β signaling pathways. For example, TGF-β, which exists in multiple isoforms such as TGF-β1, TGF-β2, and TGF-β3, plays a complex role in cancer. Initially, TGF-β can act as a tumor suppressor in normal and early stage cancer cells by inhibiting cell proliferation and inducing apoptosis. However, in advanced cancers, it switches to a tumor-promoting factor, contributing to processes like EMT, angiogenesis, and immune evasion. MUC1, with its aberrant expression in cancer, can enhance the tumor-promoting aspects of TGF-β. It might interfere with the normal regulatory mechanisms of TGF-β signaling, perhaps by influencing the phosphorylation of key components in the TGF-β pathway or by modulating the interaction between TGF-β receptors and their downstream effectors (159–161).

The TME also encompasses interactions with fibroblasts, where mucins can influence the production of growth factors and cytokines that alter the behavior of surrounding cells. For instance, MUC1 deficiency in fibroblasts has been associated with increased production of EGFR ligands, enhancing EGFR signaling and further facilitating lung carcinogenesis (139). This intricate feedback loop demonstrates how mucins regulate the cross-talk between tumor cells and the stroma, affecting the overall tumor dynamics. Extracellular vesicles (EVs) derived from both lung cancer cells and activated mast cells are another crucial aspect of mucin interactions in the TME. These EVs can carry tumor-promoting microRNAs (miRNAs), affecting gene expression in recipient cells, including immune cells and tumor cells (162). Such intercellular communication is vital in cancer progression and emphasizes the role of mucins as not merely structural components but active participants in signaling pathways that drive tumorigenesis.

The immunosuppressive effects of MUC1 in the TME can also be mediated through its influence on other mucins and cancer-related pathways. For example, interactions between different mucins—such as MUC5AC and other mucins—indicate a network where these proteins might cooperate or antagonize each other’s functions, further complicating the TME. MUC5AC has been implicated in lung cancer cell metastasis and growth, and its knockdown has led to decreased migration of cancer cells and reduced tumor-promoting signaling pathways, particularly via integrin-mediated pathways which are essential for cancer cell adhesion and motility (67).

MUC4, characterized by its extensive glycosylated extracellular region, has the ability to conceal immunogenic antigens on the cell surface, thus shielding tumor cells from immune system recognition (126). The MUC4 protein contains multiple identical tandem repeat units, which are crucial for its structural and functional properties. MUC4 comprises distinct domains that contribute to its diverse functionality. Its structure is highlighted by: N-Glycosylated Region: Essential for protein folding and stability, this region carries crucial N-glycan chains at the amino terminus; Core Region: Harboring conserved sequences and functional domains, it serves as the primary active segment of MUC4; C-Terminal Region: Incorporates signal peptides and transmembrane helices, guiding MUC4’s positioning and function; Glycan Chain Diversity: Including O-glycosylation and Sialylation, enhancing its complexity and potentially modulating function; Post-Translational Modifications: Acetylation and phosphorylation significantly impact MUC4’s activity and behavior; Molecular Interactions: It engages with various molecules like TGF-β and IFN-γ (interferon-γ) (123, 163, 164). The expression of the glycosyltransferase ST6GAL1 (ST6 Beta-Galactoside Alpha-2,6-Sialyltranferase 1) is closely associated with MUC4 expression and plays a role in the glycosylation process. The overexpression of ST6GAL1 in bronchial epithelial cells led to an increased expression of MUC1 and MUC4, while the silencing of ST6GAL1 resulted in a decreased MUC4 expression (144).The highly glycosylated tandem repeat (TR) domain of MUC4 has been hypothesized to impede tumor cell interaction with ECM proteins, suggesting a mechanism by which MUC4 could modulate the physical and biochemical environment of the tumor. Moreover, MUC4 has distinct extracellular and cytoplasmic tail domains that work together to promote EGFR signaling, which is essential for cancer cell behaviors such as invasion and metastasis (165).

In addition, MUC4 suppressed the growth of lung cancer cells via the modulation of the cell cycle and the GSK3β/p-Akt protein. Moreover, it influences cell invasion and metastasis via FAK activity and EMT markers (16). Furthermore, MUC4 has been shown to modulate the phosphorylation and activation of ERK, which in turn, prevents cell apoptosis. It is also involved in the activation of the c-Jun N-terminal kinase (JNK) and p38 MAPK signaling, which are necessary for cancer cell proliferation and immune evasion (166, 167). And MUC4 is capable of physically interacting with EGFR, thereby influencing its stability and activity. This interaction is crucial in sustaining oncogenic signaling within the TME, particularly as it pertains to the trans-differentiation of pancreatic acinar cells into ductal phenotypes, a process that is also indicative of lung cancer progression. MUC4’s juxtamembrane EGF-like domains are responsible for preventing EGFR ubiquitination and degradation upon ligand stimulation, which allows for persistent activation of downstream signaling pathways essential for tumor development, such as the ERK and PI3K/Akt pathways (168).

In addition, MUC4 enhances tumor cell migration and invasion by suppressing the expression of the intercellular adhesion molecule and the integrin receptor (165, 169). MUC4 also increases the activity of EGFR family proteins, which in turn activate cancer cell proliferation, growth, motility, and invasion through actin filaments (30, 170). These studies indicate that MUC4 plays a role in enhancing tumor growth by initiating various pathways that promote tumor sustenance while suppressing the apoptotic process in cancer cells (123). In the lung cancer TME, MUC4 reinforces its influence through an array of biological functions, including the promotion of inflammation and immune evasion. TAMs, particularly those polarized to the M2 phenotype, produce a microenvironment that supports CSCs generation. Studies indicate that MUC4 expression is elevated in the presence of M2-TAMs, which enhances the stemness characteristics of lung cancer cells. This suggests that MUC4 plays a vital role in promoting an inflammatory microenvironment that is conducive to CSCs generation, thereby supporting tumor malignancy and treatment resistance (156).

In MUC4 knockout cells, researchers observed the upregulation of COL4A5 (Collagen Type IV Alpha 5 Chain), SMAD6 (SMAD Family Member 6), CXCL1 (C-X-C Motif Chemokine Ligand 1), DUSP2 (Dual Specificity Phosphatase 2), and other genes, while S100A4 (S100 Calcium Binding Protein A4), PDGFRB (Platelet Derived Growth Factor Receptor Beta), CAV1 (Caveolin 1), CAV2 (Caveolin 2), and other genes were downregulated (171). Studies have shown that the expression of MUC4 is incrementally increased during the progression of pancreatic cancer and correlates with the activation of Wnt/β-catenin signaling pathways, which are known to interact with MUC4 and contribute to the modulation of the tumor microenvironment. The presence of MUC4 can enhance cancer cell migration and promote EMT, thus facilitating metastasis through increased plasticity of tumor cells (172).

**Figure 2:**
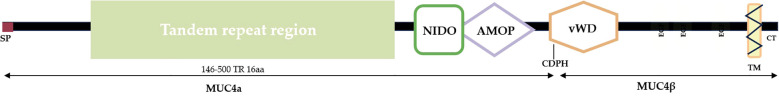
Schematic diagram of MUC4 structure. The NIDO domain, situated in the middle of the protein, may facilitate interactions with other proteins or binding to molecules. Downstream of the NIDO domain is the AMOP domain, which likely participates in protein folding, stability, and localization. At the end of the protein lies the vWD domain, potentially involved in glycosylation and modification processes. Additionally, downstream of the vWD domain are Egf-like repeats that may participate in binding to cell surface receptors (177, 178).

The ErbB receptor family, consisting of ErbB1 (HER2/neu), ErbB2 (also HER2), ErbB3, and ErbB4, interacts with MUC4. MUC4-ErbB binding triggers downstream signaling cascades, modulating cell growth, differentiation, survival, and more. The interaction may involve ErbB receptor tyrosine phosphorylation, catalyzing a biochemical sequence that elicits a targeted cellular response (173–175). Investigations into lung cancer cell lines have indicated that MUC4’s effect on ErbB signaling can lead to the activation of downstream pathways, including the MAPK and PI3K/Akt pathways. These pathways are pivotal for processes such as cell growth, survival, and motility. High expression levels of ErbB and its ligands in the TME correlate with enhanced cancer aggressiveness, highlighting the importance of MUC4’s role in the modulation of this signaling axis (176). This is crucial in tumorigenesis, as HER2 signaling is often implicated in the aggressive nature of various cancers, including lung cancer. Studies have indicated a correlation between MUC4 expression and the degree of differentiation, tumor stage, and ErbB2 expression in NSCLC. The binding of MUC4 to ErbB2 receptor tyrosine kinase involves the three EGF-like domains of MUC4β (**Figure 2**). This binding forms an oncogenic complex that promotes cell proliferation and migration and is highly expressed on the surface of cancer cells (176). The role of MUC4 in NSCLC tumor cells appears to be limited to apoptosis and the inhibition of differentiation, with no impact on the proliferation of the cells. Excessive MUC4 expression is strongly associated with poor differentiation, advanced tumor stage, and high ErbB2 expression (179). MUC4 can modulate the signaling potential of ErbB2 by stabilizing and directly interacting with the ErbB2-ErbB3 heterodimer, thereby promoting the autophosphorylation of ErbB2 (180).

Additionally, MUC4 has been found to influence the tumor-associated immune response by modulating the recruitment and activity of various immune cell types. Tumors with high MUC4 expression may show altered infiltration patterns of immune cells such as T cells and macrophages, which can contribute to a pro-tumor microenvironment (164, 181, 182). Specifically, MUC4’s role in enhancing ErbB signaling pathways is linked to creating a microenvironment that promotes the accumulation of immune suppressive cells, thereby hindering effective anti-tumor immunity. The silencing of MUC4 inhibits TGF-β1-induced EMT through the ERK1/2 pathway and induces EMT in human airway epithelial cells (158).

Overall, the role of MUC4 in lung cancer development is complex and multifaceted, involving significant interactions with various pathways and cell types within the TME. Its dual role in promoting tumor growth while also impacting immune response underscores the necessity for further research in this area, to elucidate the mechanisms through which MUC4 can be effectively targeted for cancer therapy.

The expression of MUC5AC is closely associated with tumor aggressiveness and poor prognosis in various types of cancer. In lung cancer, particularly LUAD, MUC5AC expression is significantly upregulated and correlates with tumor invasiveness, lympho-vascular invasion, tumor heterogeneity, and poor prognosis (Human lung cancer cohort study) (68). In LUAD, MUC5AC expression is notably linked to aggressive subtypes such as Invasive Mucinous Adenocarcinomas (IMA) (183). High MUC5AC expression is associated with KRAS-mutated LUAD, whereas it is lower in LUAD with EGFR mutations (183).MUC5AC-positive tumors are associated with poorer prognosis and are an independent factor for adverse outcomes.

The mechanisms underlying the role of MUC5AC in lung cancer are complex and multifaceted, involving tumor promotion, brain metastasis, cell migration, and chemoresistance (184). Specific signaling pathways activated by MUC5AC vary across studies. Some emphasize the importance of the EGFR/Ras/Raf/ERK signaling cascade (183), while others focus on the interaction between NECTIN2 and PRRC1. MUC5AC interacts with NECTIN2 to influence T-cell function and tumor angiogenesis, thereby promoting tumor immune evasion. Additionally, MUC5AC interacts with PRRC1 to enhance tumor glycosylation, which in turn boosts angiogenesis and metastatic potential (184).Overexpression of MUC5AC has been associated with cisplatin resistance in lung cancer cells. In contrast, silencing MUC5AC leads to decreased migratory capacities and increased cytotoxicity to cisplatin, suggesting that abnormal glycosylation confers resistance to therapeutic agents (67, 69).

Studies have demonstrated that MUC5AC is not only elevated in tumorous tissues but can also influence various immune processes within the TME. One of the notable findings is that MUC5AC impacts the behavior and interaction of immune cells within the TME. The interaction between MUC5AC and integrin β4 activates downstream signaling pathways by recruiting phosphorylated FAK (Y397), leading to lung cancer cell migration (67). The phosphorylation of FAK at tyrosine 397 was found to be reduced in MUC5AC knockdown cells, indicating that MUC5AC may enhance metastatic potential through integrin-mediated signaling pathways. This process is post-transcriptionally regulated by the SNHG16/mir-145 axis (185). Researchers have meticulously categorized the intricate involvement of abnormal MUC5AC secretion and production, revealing the complex role of NF-κB and IL-13–STAT6–SPDEF signaling in cell differentiation processes related to mucus secretion (19).

The pivotal role of the MUC5AC/ANXA2 signaling axis in facilitating brain metastasis from lung adenocarcinoma underscores its significance in cancer progression. ANXA2, a calcium-responsive phospholipid-binding protein, exhibits ubiquitous upregulation in diverse tumor types, notably lung malignancies, where it orchestrates pivotal functions. These include fostering angiogenesis, facilitating extracellular matrix remodeling, and serving as a crucial receptor for tissue plasminogen activators, thereby potentiating plasminogen activation and metastatic cascades (186, 187).

A notable interaction between MUC5AC and ANXA2 on cellular membranes fosters migration and colonization within cerebral niches, mediated by astrocyte-secreted CCL22. This chemokine not only triggers MUC5AC expression through the ERK1/2-SP1 signaling cascade but also binds to its receptors on lung adenocarcinoma cells, activating the ERK1/2 pathway and enhancing SP1 binding to the MUC5AC gene promoter. Intriguingly, ERK inhibitors have been found to attenuate CCL22-induced upregulation of MUC5AC and SP1, highlighting the intricate regulatory mechanisms underlying this signaling network. In summary, the MUC5AC/ANXA2 signaling pathway, modulated by CCL22, exerts a profound influence on the development of brain metastases from lung adenocarcinoma (188–191). Furthermore, the pathological effects of MUC5AC in lung cancer go beyond immune modulation. It has been reported that high mucin levels correlate with increased metastatic potential and worse overall clinical outcomes in patients, highlighting its role as a biomarker in the progression of LUAD, particularly in KRAS-mutant subtypes. Studies using genetically engineered mouse models of LUAD have shown that the depletion of MUC5AC leads to a substantial reduction in tumor growth and metastasis, underscoring its functional importance in tumor evolution and progression (192).

The MUC5B gene exhibits pronounced upregulation in LUAD, forming intricate gene networks with others, implicated in diverse functions encompassing o-glycosylation, immune system dynamics, and Golgi apparatus functions (193). These findings provide potential clues for early diagnosis of lung adenocarcinoma. Studies have revealed that MUC5B is significantly upregulated in LUAD, with a gene expression profile showing a logFC of 2.36 and a p-value of 0.01. The median OS is less than 50 months, and the hazard ratio is 1.4. These findings suggest that MUC5B may serve as a diagnostic biomarker for LUAD metastasis. Moreover, researchers have found that mutations in MUC5B are positively correlated with immune cells in the TME, such as cancer-associated fibroblasts and myeloid-derived suppressor cells (193).

Notably, the impact of MUC5B mutations on prognosis varies across different tumors. In LUAD, endometrial cancer, and bladder cancer, MUC5B mutations are associated with favorable prognosis, whereas in head and neck squamous cell carcinoma, the opposite is observed. This discrepancy may arise from differences in cellular characteristics, microenvironment, and genetic background among various tumors (193, 194). These studies elucidate the role of MUC5B in lung cancer development from multiple dimensions, including immune regulation and gene expression control. Although conclusions vary, they complement each other and deepen the understanding of the complex mechanisms underlying MUC5B’s actions. This knowledge provides a multifaceted perspective and theoretical support for lung cancer research and the development of targeted therapies. It also highlights the need for further exploration of MUC5B’s specific mechanisms in different tumor contexts and its potential therapeutic targets.

MUC6, a secretory mucin, is aberrantly expressed in various cancers, including lung cancer. Research has demonstrated that MUC6 expression is significantly elevated in LUAD, particularly in the IMA subtype. In a specific study, MUC6 expression was correlated with tumor invasiveness, lympho-vascular invasion, tumor heterogeneity, and poor prognosis. Conversely, high MUC6 expression was associated with smaller tumor size, female patients, and better prognosis. Additionally, MUC6 expression was linked to KRAS wild-type (KRAS-WT) tumors, suggesting its potential as a prognostic biomarker (71, 195).

In contrast to MUC1 and MUC21, which are strongly correlated with macrophages, MUC6 is significantly positively correlated with CD4(+) T-cell infiltration. This finding suggests potential differences in the immune microenvironment associated with these mucins. Through immunohistochemical staining, studies have indicated that the level of MUC6 expression is linked to the degree of malignancy and metastatic tendency in lung adenocarcinoma but not in lung squamous cell carcinoma (196, 197).

Investigations leveraging transplanted tumor models have conclusively shown that silencing MUC13 delays the progression of lung cancer xenografts and curbs the expression of Ki-67, a pivotal proliferation marker. Subsequent mechanistic insights have elucidated that MUC13 facilitates lung cancer advancement by stimulating the ERK/JNK/p38 signaling cascade (80).

MUC16, also known as CA125, is a large transmembrane mucin that exhibits aberrant expression in a variety of cancers, including lung cancer (198, 199). Research has shown that its expression is significantly upregulated in lung cancer, particularly in NSCLC. Moreover, elevated levels of MUC16 are associated with tumor invasiveness, lympho-vascular invasion, tumor heterogeneity and poor prognosis (200). It may impact the growth and metastasis of lung cancer cells by regulating TSPYL5 (TSPY Like 5) expression via the JAK/STAT3/GR pathway. Additionally, MUC16 can induce migration from epithelial cells to mesenchymal cells through the Src signaling pathway and can affect p53 degradation, leading to chemotherapy resistance. Through its glycan chains, MUC16 interacts with various molecules such as integrins and growth factor receptors, participating in cellular signaling, adhesion, and migration processes (201, 202). These findings shed light on the potential mechanisms underlying lung cancer progression (203). A study unveiled a pronounced elevation in MUC16 levels among patients with NSCLC upon disease progression, contrasting with a marked reduction from baseline upon treatment response. Notably, patients presenting with stage IV disease at baseline demonstrated a heightened likelihood of exhibiting elevated MUC16 levels, highlighting the potential of this biomarker to reflect disease status and response to therapy (204).

In the lung cancer tumor microenvironment, MUC16 suppresses innate immune responses through multiple mechanisms, thereby promoting tumor immune evasion. In murine models, MUC16 inhibits the cytolytic functions of natural killer (NK) cells and macrophages, mimicking its effects on human immune cells and enabling cancer cells to evade innate immune surveillance (205). Simultaneously, MUC16 binds to the Siglec-9 receptor on NK cells, inhibiting their cytotoxic function. This inhibition not only diminishes the direct killing capacity of NK cells against tumor cells but also impairs NK cell-mediated antitumor activity by disrupting immune synapse formation (206, 207). Furthermore, the extensive glycan structures of MUC16 create a physical barrier on the surface of tumor cells, hindering effective immune synapse formation between immune cells and tumor cells. This physical obstruction further restricts immune cell recognition and attack of tumor cells, providing an additional protective mechanism for tumor cells (74, 208). Additionally, aberrantly glycosylated forms of MUC16 may reduce the presentation of tumor-specific peptides via HLA-A and HLA-B molecules, enabling tumor cells to escape immune system recognition and facilitating tumor immune evasion, thus promoting tumor cell survival, proliferation, growth, and metastasis within the immune microenvironment (209).

The ability of MUC16 to activate the JAK2/STAT3 pathway exacerbates this immunosuppressive environment. The JAK2/STAT3 pathway not only drives tumor cell proliferation and metastasis but also upregulates PD-L1 expression. Moreover, the STAT3-driven transcriptional program induced by MUC16 suppresses pro-inflammatory cytokines such as IFN-γ and TLR9, thereby inhibiting both innate and adaptive immune responses. A particularly innovative aspect of the interaction between MUC16 and the lung cancer tumor immune microenvironment (TIME) is its regulation of the RNA-binding protein HuR, which post-transcriptionally stabilizes oncogenic transcripts such as c-Myc. This mechanism promotes tumor progression while indirectly inhibiting immune surveillance by altering the expression of immunomodulatory genes (149, 210).

In patients with LUAD, MUC16 mutations are significantly associated with higher TMB. High TMB is typically linked to stronger immunogenicity, as nonsynonymous mutations generate neoantigens that can be recognized by the immune system to elicit an antitumor immune response (211, 212). In another study, MUC16 was found to be significantly upregulated in ovarian cancer cells, and delivery of MUC16 via dendritic cell (DC)-based vaccines could stimulate CD8+ cytotoxic T lymphocytes (CTLs) to eliminate tumor cells, highlighting the close association between MUC16 and DC-mediated immune responses (213). However, even in the presence of high TMB, T cell activation may be limited if antigen presentation mechanisms are impaired. Effective T cell responses via neoantigens are likely only when MUC16 mutation burden is extremely high and accompanied by an intact antigen presentation mechanism.

In the context of the TME with MUC16 mutations, the expression of immune checkpoint molecules PD-L1, LAG3, PDCD1, and SIGLEC15 is significantly upregulated. This immunosuppressive microenvironment may counteract the immune advantages conferred by high TMB, leading to poor prognosis. Furthermore, in the tumor microenvironment of high-risk LUAD patients, although the abundance of CTLs and pro-inflammatory immune signals increases, the functions of these immune cells may be constrained by MUC16-mediated immunosuppression (211, 214, 215).

Moreover, in air pollution-related lung cancers, MUC16 mutations have been shown to be particularly prevalent, indicating that environmental factors may influence its expression and, subsequently, the tumor-associated immune responses. The presence of increased MUC16 mRNA levels in lung cancer tissues from heavily polluted areas correlates with poor prognosis, further establishing a link between MUC16 expression, environmental carcinogens, and immune system interactions in lung cancer development (216, 217).

While MUC16 as a standalone biomarker does not significantly impact survival outcomes in NSCLC patients, albeit with a discernible trend, its integration with IL-24 into a biomarker panel reveals a substantial improvement in OS. This finding underscores the potential of leveraging the MUC16-IL-24 combination in screening protocols to enhance the efficacy of early detection for NSCLC (218).

In NSCLC patients receiving EGFR TKIs, MUC17 is downregulated in drug-resistance-acquired cells. Moreover, MUC17 is reported to increase NF-κB activity through the enhancement of DNMT1/UHRF1 complex-mediated promoter methylation. This implies that MUC17 could be an epigenetic biomarker for measuring resistance to EGFR-TKIs in drug-resistance-acquired cells. In addition, MUC21 upregulates the expression of Bcl-2 and downregulates the expression of BAX and cleavage of caspase-3 through the activation of the JAK2/STAT3 signaling pathway, preventing cell apoptosis. Related study shows that the deglycosylation of MUC21 diminishes its anti-apoptotic properties and that sialidase treatment reverses this effect. These considerations point to the possibility of employing both MUC17 and MUC21 as further targets for studying drug resistance and possible approaches to addressing this issue in NSCLC patients receiving EGFR-TKI (88, 124, 219–221).

Based on what we know so far, the changes in the level of mucins can be considered an auxiliary diagnostic criterion in the case of lung cancer. Therefore, for the purpose of establishing new strategies for therapeutic interventions, understanding the regulatory mechanism of the mucin family and its intricate association with the development of lung cancer becomes imperative. For future research on this subject, creating a detailed map of the lesions and the microenvironment where they exist may be helpful. This approach could reveal more information of the dynamic changes in the lesions in lung cancer and may have implications for earlier diagnosis and treatment of the disease. In conclusion, further studies in this area can help to enhance our understanding of the mucin involvement in lung cancer; discover novel therapeutic targets for the treatment of lung cancer; and most importantly, contribute to betterment of patient prognosis.

### 3.2 Prognostic value of mucins in patients with lung cancer

The relationship between the presence of mucins and the occurrence of lung cancer is significant. Both enhance survival and proliferation of lung cancer cells by maintaining cell division and death. These mucins are also responsible for the cell cycle and assist the cells in avoiding apoptosis, and hence, they are responsible for the formation of tumors. Moreover, mucins are implicated in the invasion and metastasis of lung cancer cells since they affect the adhesion and motility of cancer cells, thus enhancing the ability of cancer cells to invade surrounding tissues and organs (222).

Of all the mucins, MUC1 has been found to be significantly linked to poor survival and prognosis. Inappropriate levels of MUC1 cause a disruption in the epithelial cell polarity and change the downstream signals through the cytoplasmic domain of MUC1, leading to an enhanced malignant potential of cancer (223). In these mucin family members associated with lung cancer.

The significant upregulation of MUC1 expression in LUAD has important clinical implications, as it may serve as a diagnostic marker and guide personalized treatment strategies for this subtype of lung cancer. MUC1(+) tumors are more likely to have lymph node metastasis and cleaved caspase-3 expression, as well as a larger tumor diameter than those that are MUC1(-). Thus, evaluating the prognostic significance of MUC1 in lung cancer is a very significant task. In addition, poor-prognosis tumor types also exhibit nonpolarized MUC1 expression patterns, further illustrating the critical role of MUC1 in the prognostic assessment of lung cancer (224).

Likewise, the critical role of MUC4 in tumor biology must be acknowledged and considered. MUC4 has gained increasing recognition for its involvement in tumor progression and immune evasion mechanisms. Its expression levels exhibit significant variation across different types of lung cancer; studies have demonstrated that elevated MUC4 expression is associated with adverse clinical outcomes in lung adenocarcinoma and lung squamous cell carcinoma. Findings further indicate that MUC4 is closely linked to lymphatic and vascular invasion, thereby positioning it as a potential biomarker for aggressive tumor behavior. Specifically, an analysis of a patient cohort revealed that high MUC4 expression correlates with reduced overall survival (OS) rates among lung cancer patients, underscoring its prognostic significance (181, 225). Additionally, evidence showing an inverse correlation between MUC4 expression and survival outcomes further demonstrates that its overexpression negatively impacts cancer progression (123, 226).

Another study also showed that a high MUC5B expression is prognostic for poor survival in lung cancer patients, particularly those with IMA (23, 227). MUC5B is controlled by many TFs like NKX2-1, SPDEF, and FOXA3, and any distortion in the expression of these TFs directly impacts the IMA (228). Moreover, certain mutation sites of the MUC5B gene are significantly correlated with the response and survival rate of NSCLC patients after radiotherapy, which will offer valuable implications for the individualized treatment of lung cancer. MUC5AC is also an important gene involved in the development of IMA, and IMA samples with a high expression of MUC5AC are commonly associated with a poor prognosis (229). The expression of MUC5AC is also under the regulation of a series of regulatory factors including DNA methylation. Thus, if the genes that are involved in the development of lung cancer are better regulated, then there will be more control over the growth of the disease (230).

The expression pattern of MUC6 gives new insight regarding its use as a prognostic factor for IMA patients (231). However, MUC6 is not detected in normal lung tissues, while it is overexpressed in IMA and related to certain IMA types. The results suggest that MUC6 is highly expressed in IMA, and its high expression is associated with better prognosis, which adds it as a new marker to the classification and prognosis of IMA (195).

MUC16 can also be used as a biomarker to assess the prognosis of patients with lung cancer, as suggested by data obtained throughout the course of a study by Zhang et al. (232). Pre-chemotherapy MUC16 levels correlate strongly with patient outcomes and treatment responsiveness, with lower baseline values predictive of extended progression-free survival, suggesting the biomarker’s prognostic value in NSCLC management strategies (233). Other researchers have developed an immune prognostic model (IPM) to ensure that MUC16 is commonly mutated in LUAD with a rate of 43.4% and comes the third rank, significantly correlating with a high TMB (211). Researchers have found that MUC16(+) is an independent risk factor for poor survival in some cancer patients, and the OS of patients with MUC16(+) cholangiocarcinoma (27.4 months) is significantly lower than that of patients with MUC16(-) (56.1 months) (234). The same conclusion was found in NSCLC, where MUC16(+) patients had a median survival of 18.84 months, and expression levels were inversely correlated with survival (235).

However, present studies about the correlation of mucin with the survival of the patients with lung cancer have certain limitations. Although a strong mucin expression is associated with poor prognosis in patients with lung cancer, the molecular mechanism has not been elucidated yet and further investigations are required to clarify the molecular basis. Moreover, given the diversity of lung cancer, the association between mucin and the outcome of patients may not be the same for all subtypes of the disease. Despite promising findings, significant challenges remain in accurately determining the association between mucin expression and clinical outcomes across diverse lung cancer subtypes. These challenges include the heterogeneous nature of lung cancer, the complexity of mucin signaling pathways, and the need for larger, well-controlled studies. Addressing these challenges will be crucial for the development of mucin-targeted therapies.

Hence mucins have predictive values for the prognosis of lung cancer, but the specific molecular mechanism and clinical application of mucin remain to be further explored. Further studies should be undertaken to explain the exact function of mucin in the development of lung cancer in order to enhance the utilization of mucin as a diagnostic marker or prognostic indicator for lung cancer and to provide a theoretical foundation for the individualized treatment of lung cancer.

## 4 Breakthroughs in the use of mucins as therapeutic targets for lung cancer

The field of using mucin as a therapeutic target for lung cancer is making rapid progress, with significant advancements in fundamental research and notable developments and challenges in clinical research. Randomized controlled trials are currently under way to determine whether novel treatments designed to address mucin dysregulation are effective; the results of some of these trials are positive in specific instances. However, challenges still need to be overcome, for instance, how to deliver therapeutic agents specifically to tumor tissues and how to minimize the adverse effects of the treatment (236, 237).

### 4.1 Limitations of traditional targets

Lung cancer, a highly lethal malignant tumor, is typically treated with surgical intervention, chemotherapy, radiotherapy, and targeted therapy (238). Over the past few years, targeted therapy has been identified as an essential strategy for lung carcinoma since it focuses on the molecular markers found on cancerous cells (239). However, existing approaches to selectivity remain problematic today.

Current targeted therapies are primarily based on a small panel of gene mutations like EGFR and ALK, which are detectable in a small proportion of lung cancer patients in the clinic. By inference, currently available targeted therapies for lung cancer may not be effective for most patients (240). Furthermore, the same treatment is often prescribed to address a particular target, but the response of patients is quite diverse (241). The major cause of this difference is the heterogeneity of lung cancer cells, which is apparent not only in the differences in their gene expression profiles but also in the differences within their tumor microenvironment (242).

In addition, many drugs in targeted therapy often face the problem of drug resistance. This includes typical clinical scenarios, including platinum drug resistance and EGFR TKI resistance (243, 244). During the course of treatment, the cancer cells may undergo genetic changes that make them resistant to these targeted drugs (245). The emergence of drug resistance not only reduces the long-term efficacy of targeted therapy but also explains why it is costly.

In conclusion, the current targeted therapies for lung cancer have been found to be partly effective. Therefore, investigations into new targets for treatment, for instance mucins, have the potential to bring about significant progress in the management of lung cancer. Improving the quality of treatment and extending the range of interventions for patients with lung cancer would thus be possible.

In the search for new targets for therapy, scientists have also turned to immunotherapy in tackling lung cancer. Immunotherapy is a type of cancer treatment that uses a person’s immune system to fight cancer cells (246). This approach has been found to have some efficacy in some patients, increasing survival and reducing side effects compared with conventional therapy (247).

Moreover, developments in precision medicine and genetic testing have enabled treating patients with more targeted treatment plans (248). For instance, through sequencing patient’s tumors, doctors can see the different mutations that exist in a tumor and can develop new treatments that can target these mutations, thereby enhancing patient survival (249).

Further research and clinical trials, which would help expand on our knowledge of these new therapies and their compatibility with other targeted therapies, should be funded. As long as people remain committed and eager to find new methods of fighting the disease, the possibility for further success remains possible.

### 4.2 Advances in mucin as novel therapeutic targets

Mucins could be of interest for the development of new molecular therapies for lung cancer because of their possibilities as diagnostic and therapeutic biomarkers, their impact on tumor progression, and their link with patient survival. These biological molecules can be considered to have benefits over traditional therapeutic strategies due to the lower incidence of side effects and drug resistance. Although the role of mucins in lung cancer has not been fully understood due to the complex and multifaceted nature of the connection, the immune map of mucin-dominant precancerous lesions and the microenvironment surrounding them is anticipated to open a new perspective on and a new way of diagnosing and treating lung cancer lesions. Several studies have been conducted to support this research (35, 49, 171, 220, 250).

The opportunity of using mucin as the target of treatment of lung cancer becomes clear in several aspects. First, the abnormal expression of mucins in lung cancer cells acts as the basis for the identification and treatment of this disease. The expression level of mucins in lung cancer cells is higher/lower than that of normal cells, which opens up the possibility of using mucins in the diagnosis and treatment of lung cancer.

Second, mucin is crucial for the tumorigenesis and development of lung cancer, which indicates its potential as a therapeutic target. The overexpression of mucin in lung cancer cells not only stimulates its proliferation and invasion but also enhances its ability to resist apoptosis, which poses a problem for the treatment of the disease. Thus, current therapies focusing on mucin can be considered promising for increasing the effectiveness of treatment.

Additionally, mucin has been closely associated with the survival of patients with lung cancer, which makes it a potential target for enhancing patients’ outcomes. High levels of mucin were observed to be more common in lung cancer patients and may be related to shorter survival (251).

Furthermore, mucin is a relatively newly identified target for therapy, and we envision that treatments using this target may have fewer side effects and be safer than many other targets. Such a treatment is also associated with fewer issues with drug resistance, making it a promising new avenue for attacking lung cancer.

Currently, further research into targeted therapies for lung cancer concentrates on mucin, including anti-mucin therapy and mucin modulators. However, these studies have faced some limitations: insufficient information regarding the regulatory mechanisms of mucin and the lack of a standard method to assess the therapeutic benefits of targeting mucin (252).

Further research should prioritize investigating the specific mechanisms through which mucin affects lung cancer, as well as its potential side effects and interactions with other treatments. Clinical trials will be crucial for determining the optimal dosage and administration of mucin-targeted therapies, as well as their long-term impact on patient outcomes. Additionally, exploring the potential for combination therapies involving mucin-targeted drugs and other treatment modalities, such as chemotherapy or immunotherapy, will be important.

Concurrently, developing reliable diagnostic tools for identifying patients who are most likely to benefit from mucin-targeted therapies will be essential. This may entail the discovery of specific biomarkers or genetic signatures capable of predicting a positive response to treatment.

In general, while targeting mucin for lung cancer therapy shows promise, further research is required to fully comprehend its potential benefits and limitations. Collaborations among researchers, clinicians, and pharmaceutical companies will be crucial in advancing this field and ultimately enhancing the outcomes of patients with lung cancer.

However, some steps in the investigations of mucins as potential targets for therapeutic intervention in lung cancer have been encouraging. For example, anti-mucin treatments have been discovered, like monoclonal antibodies and small molecule inhibitors, such as NCT00157209, NCT02140996, NCT04695847, NCT04020575, are mucin-related clinical studies registered with ClinicalTrials.gov. Furthermore, some scholars are considering the application of mucin modulators to control the synthesis and activity of mucin as a lung cancer therapy (**Tables 4**, **5**).

**Table 4:** Summary of clinical development status of mucin-targeted therapies for NSCLC.

		Research status	Tumor type	Source
Monoclonal antibody	PankoMab-GEX(IgG1)	Phase I clinical trial	NSCLC	(253)
	DS6	Preclinical study	NSCLC	(254)
	SAR566658	Preclinical study	NSCLC	(254)
	DF3	Preclinical study	NSCLC	(255)
	KL-6	Preclinical study	NSCLC	(256)
	HMFG1	Preclinical study	NSCLC	(257)
Antibody coupled ADC therapy	16A+MMAE	Preclinical study	NSCLC	(258)
	anti-MUC1-C/NPs	Preclinical study	NSCLC	(259)
	mAb 3D1-MMAE	Preclinical study	NSCLC	(260)
	mJAA-F11	Preclinical study	NSCLC	(261)
	CH129	Preclinical study	NSCLC	(262)
	GT-00AxIL15	Preclinical study	NSCLC	(263)
	Qbeta-MUC1	Preclinical study	NSCLC	(264)
Vaccine	M-1-PL-co-GA-PEG-sHA-NPs	Preclinical study	NSCLC	(265)
	TG4010	Phase III clinical trial	NSCLC	(266)
	Tecemotide	Phase III clinical trial	NSCLC	(267)
	Ad-sig-hMUC1/ecdCD40L	Phase I clinical trial	NSCLC	(268)
	MUC1-Vax	Preclinical study	NSCLC	(269)
	CpDV-IL2-sPD1/MUC1	Preclinical study	NSCLC	(270)
	CV9202	Phase IV clinical trial	NSCLC	(271)
ncRNA	miR-145	Preclinical study	NSCLC	(272)
	miRNA-29b	Preclinical study	NSCLC	(273)
Protein inhibitor	GO-203	Preclinical study	NSCLC	(274)
Combined treatment	bevacizumab + tecemotide	Phase III clinical trial	NSCLC	(275)

**Table 5:** Examples of mucin-based therapeutic strategies for NSCLC.

Target	Content	Tumor type	Source
Tan CAR-T	Constructe a bivalent tandem CAR-T (Tan CAR-T), which can simultaneously target MUC1 and PSCA	NSCLC	(276)
MUC-1/CD3 BsAb	combine EpCAM/CD3 BsAb and MUC-1/CD3 BsAb to target both EpCAM and MUC-1	NSCLC	(277)
epitope APDTRP	Be recognized by multiple anti-MUC1 antibodies and then activate tumor antigen-specific cytotoxic T lymphocytes	NSCLC	(278)
16-amino-acid MUC1 peptide	Couple to keyhole limpet haemocyanin (KLH) (BP16-KLH) plus DETOX adjuvant elicited evident class-I restricted CTL activation	NSCLC	(279)
25-amino-acid VNTR MUC1 peptide	Liposomal vaccines targeted to the mucinous carcinoma-associated glycoprotein MUC1—L-BLP25	NSCLC	(280)
MUC1 glycopeptide	Consist of MUC1 glycopeptide antigen and a T-cell epitope for the induction of a highly specific humoral immune response	NSCLC	(281)

Here, we highlight the potential of these approaches through several representative clinical trials. NCT00157209, a Phase IIb randomized controlled trial, evaluated the safety and efficacy of Tecemotide, a MUC1-targeting vaccine, in combination with best supportive care (BSC) for patients with stage IIIb or IV NSCLC who had stable disease or response after first-line therapy (chemotherapy ± radiotherapy). Although the overall trial did not meet its primary endpoint of OS, a subgroup analysis revealed a significant improvement in median OS for patients receiving Tecemotide plus BSC compared to BSC alone (30.8 months vs. 20.6 months, HR = 0.78, p = 0.016) among those who had undergone concurrent chemoradiotherapy, suggesting the potential of MUC1 vaccines in specific patient populations.

NCT04695847 is a Phase I study assessing the safety and preliminary efficacy of M1231, a bispecific antibody-drug conjugate (ADC) targeting MUC1 and EGFR, in patients with metastatic solid tumors, including NSCLC. Utilizing Sutro’s non-natural amino acid conjugation technology, M1231 links the Hemiasterlin toxin to a bispecific antibody.

NCT02587689, a Phase I/II trial, investigated the use of anti-MUC1 chimeric antigen receptor T-Cell Immunotherapy (CAR-T) cells for the treatment of MUC1-positive advanced solid tumors, including NSCLC, enrolling 20 patients. Post-infusion, 11 patients achieved stable disease, while 9 experienced disease progression. However, symptom improvement was noted in all patients, with no grade 3 or higher toxicities or cytokine release syndrome (CRS).

NCT04020575 features the second-generation CAR-T therapy (HUM NC2-CAR 22) developed by Minerva Biotechnologies. This therapy, incorporating a CD3-ζ signaling domain mutation (1XX mutation), significantly extends T cell survival and enhances the killing capacity against low MUC1-expressing tumor cells. Demonstrating durable responses in breast cancer models, it is now being expanded to a lung cancer cohort, with preliminary data indicating persistent CAR-T cells in the body and partial responses in some patients.

AICAR (5-Aminoimidazole-4-Carboxamide Ribonucleoside), an endogenous purine metabolite, has been found to inhibit lung tumor growth by targeting MUC1 (282). Furthermore, the construction of an MUC1-DNA vaccine expressing 42 tandem repeats inhibited the growth of MUC1-expressing tumors in BALB/c mice following an injection of pcDNA-MUC1. Dual-specificity CAR-T cells, known as Tan CAR-T cells, targeting MUC1 in combination with an anti-PD-1 antibody showed promise for the treatment of NSCLC (283). Study has elucidated the potent effect of DCs pulsed with WT1 and/or MUC1 peptide vaccines in prolonging survival among patients diagnosed with advanced NSCLC, underscoring their therapeutic potential (284).

Additionally, the L-BLP25 vaccine, which consists of a 25 amino acid sequence (STAPPAHGVTSAPDTRPAPGSTAPP) derived from MUC1, the BL25 fat peptide, the TLR4 (Toll-like receptor4) agonist monophosphoryl lipid A (MPLA), and three lipid A adjuvants, has been shown to activate peripheral blood lymphocytes and to induce strong Cytotoxic T-Lymphocyte (CTL) responses in Phase III clinical trials involving NSCLC patients (276, 285).

In a case report, a patient with advanced LUAD received DC vaccines (loaded with WT1 and MUC1 antigens) in combination with erlotinib. The treatment led to a 65.7% reduction in tumor volume, with no recurrence within 587 days. Mechanistically, the vaccine induced a MUC1-specific CD8(+) T cell response, while erlotinib enhanced immune cell infiltration by inhibiting the EGFR signaling pathway (286).

Synthetic glycopeptide vaccines, such as ONT-10, mimic tumor-specific Tn antigens to induce high-affinity IgG antibodies. Preclinical studies have shown that the efficacy of the vaccine is associated with the glycosylation level of MUC1. Tn antigen-modified vaccines significantly inhibited tumor growth in MUC1 transgenic mice (287).Self-adjuvant glycopeptide vaccines (MUC1-Tn-Pam3CSK4) enhance immune responses via TLR2 agonists, inducing robust humoral and cellular immunity in tumor-bearing mice without the need for exogenous adjuvants (193).

TG4010 is an MVA-MUC1-IL2 vaccine. Researchers evaluated the efficacy of TG4010 in combination with chemotherapy in a Phase II trial for advanced NSCLC. Patients were randomly assigned to receive either TG4010 plus cisplatin/vinorelbine or sequential treatment (TG4010 monotherapy followed by chemotherapy upon progression). The combination group achieved an objective response rate (ORR) of 29.5%, with desirable disease control and MUC1-specific immune responses. The median OS was 12.7 months, and the safety profile was manageable (288).

## 5 Conclusion

Overall, the significance of mucins in lung cancer has attracted increasing attention due to their potential role in the development, progression, and metastasis of tumors. As research on the role of mucins in lung cancer continues, considering the potential implications for clinical practice will be important. The profound understanding of the specific roles played by different mucin family members in lung cancer holds immense potential for revolutionizing the diagnosis, prognosis, and treatment of this devastating disease. By unraveling the intricate web of mucin signaling pathways, researchers can pave the way for the development of highly personalized and effective therapies that will ultimately improve patient outcomes.

In conclusion, ongoing research into the role of mucins in lung cancer has the potential to significantly impact clinical practice by providing new tools for diagnosis, prognosis evaluation, and treatment. This could ultimately bring about improved outcomes for patients with this challenging disease, as advancing our understanding of the complex disease could result in personalized treatment strategies.
